# Extracorporeal membrane oxygenation: the MOTOR of cytokine production?

**DOI:** 10.1186/cc12065

**Published:** 2013-03-19

**Authors:** Y Hara, O Nishida, T Nakamura, S Uchiyama, J Shibata, C Yamashita, M Yumoto, Y Shimomura, N Kuriyama, N Yasuoka, M Ito, K Kawata, S Hayakawa, S Yamada, T Miyasho, K Moriyama

**Affiliations:** 1 Fujita Health University School of Medicine, Toyoake, Japan; 2 Shino Corporation, Tokyo, Japan; 3 Rakuno Gakuen University, Ebetsu, Japan

## Introduction

The usefulness of extracorporeal membrane oxygenation (ECMO) is being rediscovered in the wake of the pandemic of H1N1 influenza. However, it has been reported that patients who received ECMO often developed virus-associated hemophagocytic syndrome (VAHS), compared with those without ECMO support. Although there is ample evidence that extensive cytokine activation is a key factor in VAHS, ECMO itself could be a potential trigger to exacerbate the pathology by amplifying cytokine activation. In this study, we investigated whether mediators such as cytokines may be produced by ECMO.

## Methods

Patients with severe respiratory failure who were placed on ECMO were enrolled between June and July 2012. This study was approved by the ethics committee. Blood specimens were drawn from the blood circuit at the inlet of the centrifugal pump (before) and outlet of the hollow fiber oxygenator (after) at a frequency of three to four times per day. Blood IL-1β, IL-2, IL-4, IL-5, IL-6, IL-7, IL-8, IL-10, IL-12(p70), IL-13, IL-17, G-CSF, GM-CSF, IFNγ, MCP-1, MIP-1β, and TNFα were measured globally using a multiplex cytokine bead array system (Bio-Plex; Bio-Rad, Tokyo, Japan). HMGB1 was measured using an ELISA kit (Shino-Test, Tokyo, Japan).

## Results

Two patients with interstitial pneumonia were studied. The ECMO system consisted of a Rotaflow Centrifugal Pump (Maquet Japan, Tokyo, Japan), a Biocube TNC coating 6000 (NIPRO, Osaka, Japan), and a percutaneous cardiopulmonary support system (Capiox EBS; Terumo, Tokyo, Japan). The blood flow rate was 2.0 ± 4.0 l/minute. A total of 34 blood sets were collected. In most cases, blood levels of IL-1β, IL-2, IL4, IL-5, IL-12(p70), IL-13, IL-17, GM-CSF, IFNγ, and TNFα were below the detection limit and did not increase during ECMO. The other mediators were detected at the inlet (before), but no significant increase was observed at the outlet (after) (HMGB1, *P *= 0.33; IL-6, *P *= 0.12; IL-7, *P *= 0.22; IL-8, *P *= 0.43; IL-10, *P *= 0.84; MCP-1, *P *= 0.10; and MIP-1β, *P *= 0.65; Wilcoxon signed-rank test).

## Conclusion

The use of ECMO in patients with severe respiratory failure did not induce systemic inflammatory changes. These observations are preliminary, but may nevertheless have important implications for the future management of patients with severe infections.

